# Views on Everyday Life and Reflections on Future Death and Dying Among Older Persons Residing in Nursing Homes: A Meta‐Ethnographic Synthesis

**DOI:** 10.1111/opn.70097

**Published:** 2026-08-11

**Authors:** Peter Lundberg, Jane Österlind, Malin Olsson

**Affiliations:** ^1^ Department of Health Care Sciences Marie Cederschiöld University Stockholm Sweden

**Keywords:** death and dying, dignity, everyday life, experiences, meta‐ethnographic synthesis, nursing home, older persons

## Abstract

**Introduction:**

Life issues relating to identity, meaning and dignity are inherent to human existence and remain significant throughout the entire lifespan. To meet the needs of older persons residing in nursing homes, it is essential to understand their views on everyday life, particularly regarding the proximity of death, reflections on future death and dying, and the potential for meaning in everyday life. However, these views have received limited attention in research. The aim of this study was therefore to explore older persons' views regarding everyday life and reflections on their future death and dying while residing in nursing homes.

**Methods:**

A meta‐ethnographic synthesis was conducted by searching CINAHL, PubMed and PsycINFO supplemented with manual searches, guided by the SPIDER tool. The synthesis focused on peer‐reviewed empirical research papers published in English, between 1 January 2000 and 7 April 2025.

**Results:**

Eighteen studies were identified and analysed based on older persons' expressed experiences and views on everyday life in nursing homes. Studies with proxy perspectives or mixed‐method designs were included; however, only qualitative data representing older persons' own views were extracted and analysed, primarily from participants able to engage in interviews. Three analytic themes were formulated: *Loss of the Familiar World, Trying to Maintain Personal Identity*, *Death as Close and Tangible*. An overarching metaphor was synthesised: *Maintaining and Shaping a Living Space for a Visible Future*.

**Conclusions:**

Living in a nursing home involves an everyday life marked by losses and an ongoing awareness of future death and dying. At the same time, older persons actively strive to uphold identity, personal dignity and meaningful relationships. Understanding these processes is essential for supporting older persons in everyday nursing care.

**Implications for Practice:**

Nursing staff should make time for regular conversations about resident' lives and concerns, including thoughts about death and dying, and support activities that help them maintain their sense of self.

## Summary

**What does this research add to existing knowledge in gerontology?:**

- Older people in nursing homes actively exercise agency in expressing their identity. These actions reflect a profound effort to preserve personal dignity.
- Supporting older people in maintaining identity and a sense of meaning through everyday self‐expression, including in the face of death, is closely intertwined with preserving dignity and meaningful connections.
- This meta‐ethnographic approach provides a synthesised understanding that extends beyond individual studies on the complexity of the personal dignity of older people residing in nursing homes.

**What are the implications of this new knowledge for nursing care for and with older adults?:**

- For older people in nursing homes to live a dignified life, it is essential that nursing staff recognise and support each resident's personal identity in everyday care and daily activities.
- Nursing staff must acknowledge that an older person's contemplation of death is a complex and nuanced phenomenon integral to their identity formation, and that appreciating this can support their dignity.
- Supporting dignity requires staff to ensure flexibility in routines, create daily opportunities for conversation and shared activities, and actively engage with residents to support identity through meaningful, ongoing relationships.

**How could the findings be used to influence practice, education, research, and policy?:**

- The normative view of dignity as identity encourages a shift beyond simply addressing basic human needs, towards supporting a meaningful life enriched by connection and authentic relationships.
- Nursing home policies should explicitly recognise the dignity of identity as a core value, ensuring time, staffing and organisational conditions that enable relational and person‐centred care.
- Education and staff training should include structured reflection on identity, dignity and ageing and existential dialogue in everyday care.

## 1 Introduction

With age, views on everyday life and reflections on approaching death and dying may shift due to accumulated life experiences, relationships and life challenges (Erikson 1982; Pinquart 2002). Maintaining identity, coping with losses, creating meaning in everyday life, preserving dignity, navigating social relationships and confronting the proximity of death and dying are fundamental human concerns across the lifespan (Yalom 1980).

Although global life expectancy has increased, this is not necessarily associated with functional decline; many older people experience prolonged good health (World Health Organisation 2015). However, it is common for older persons to relocate to residential care facilities, such as nursing homes (NHs), due to physical or other impairments (Hakimjavadi et al. 2025). Older persons residing in NHs have personal experiences and views on everyday life, particularly in relation to the proximity of death and dying and how to shape a new chapter of life. However, in a literature review, Rahm Hallberg (2004) argues that older persons' own perspectives on death, dying and creating meaning in everyday life are often neglected in research.

Creating meaning in everyday life is essential for living a dignified life (Saarelainen et al. 2022). Although frequently used, the concept of dignity remains ambiguous (Macklin 2004; Killmister 2010), yet it is central in healthcare and in some countries legislated (Barroso 2012). From a theoretical perspective, Nordenfelt (2021) defines it as self‐respect rooted in autonomy and relationships—known as the *dignity of identity*, which may be challenged by illness, decline, ageing, or the actions of others. These theoretical concerns are reflected in empirical contexts; Beck et al. (2012) demonstrate that task‐oriented care in NHs may eclipse residents' existential needs, thereby illustrating how dignity can be undermined in everyday practice.

Previous review studies have addressed quality of life, dignity, hope, end‐of‐life care and experiences of care in NHs, including within models of positive ageing (Clancy et al. 2021; Greenwood et al. 2018; Otto et al. 2023; Pinto et al. 2023; Rodríguez‐Martínez et al. 2023; Vaismoradi et al. 2016). However, older persons' views on creating meaning in everyday life and reflections on approaching death and dying while residing in NHs remain largely unexplored.

Older persons may perceive everyday life in NHs as restricted and unfamiliar, often due to losses in bodily or cognitive abilities that lead to their relocation (Kingston et al. 2017). Adjusting to the new environment often requires support for self‐worth and integrity (Panthi 2023; Sun et al. 2021). Their most expressed needs concern attentive and genuinely caring staff, along with opportunities for social interaction (Schweighart et al. 2022). Meaningful social affiliation is essential for dignity and connectedness, even under restrictions, as highlighted empirically in advanced dementia care (Melander et al. 2017). These experiences can be understood through the concept of at‐homeness (Öhlén et al. 2014), which captures how well‐being, identity and meaning are shaped in the interplay between person, place and relationships in later life.

Meeting the needs of older persons residing in NHs is challenging due to tensions between individual and collective considerations (Vaismoradi et al. 2016). Eldh et al. (2016) highlight the benefits of reciprocal relationships, where care involves both assistance and valuing residents' life experiences. To uphold dignity, staff must see each person as unique and involve them in decisions to support autonomy (Šaňáková and Čáp 2019), which requires ethical awareness, communication, cooperation and tolerance within time constraints.

Older persons at risk of dependency are often stereotyped as vulnerable and a financial burden to society, a phenomenon Binstock (1985) termed compassionate ageism, which can threaten identity in older persons residing in NHs (Kagan and Melendez‐Torres 2015). Such stereotyping may also harm health (Levy 2009). Counteracting it requires understanding residents' perceptions to safeguard well‐being, dignity and identity.

Older persons are a heterogeneous group, and understanding their views on past experiences, present circumstances and remaining time is essential. There is a lack of studies, particularly meta‐syntheses, addressing the views of older persons residing in NHs on everyday life and their reflections on their future death and dying. Deeper insight into these views can inform healthcare professionals and families. This meta‐synthesis aims to fill this gap by exploring the views of older persons residing in NHs on everyday life and their reflections on future death and dying. Identity, loss, meaning, dignity, relationships and death and dying are particularly salient for older persons residing in NHs. The synthesis is theoretically informed by the concepts of dignity of identity, at‐homeness and compassionate ageism, which together provide an interpretive framework for understanding how everyday life, identity and reflections on future death and dying are shaped—and potentially challenged—in NH contexts.

## 2 Aim

The aim was to explore older persons' views regarding everyday life and their reflections on their future death and dying while residing in nursing homes.

## 3 Methods

### 3.1 Design

To synthesise research findings, we used a meta‐ethnographic approach inspired by Noblit and Hare (1988), and the systematisation generally followed the eMERGe reporting guidance (France, Cunningham, et al. 2019; France, Uny, et al. 2019). Meta‐ethnography is a widely used qualitative methodology in health and social care research (Bondas and Hall 2007; France, Cunningham, et al. 2019). It involves seeking explanations for social and cultural phenomena based on the perspectives that emerge from the persons under study. It entails a reciprocal understanding of the interrelationships within the studies, aiming to develop new overarching concepts, theories and models. This goes beyond merely summarising study results. The research process followed seven phases according to France, Cunningham, et al. (2019); France, Uny, et al. (2019) although the process was iterative. The seven phases are: (1) getting started, rationales for using meta‐ethnography; (2) deciding what is relevant, data collection—selection and search outcomes; (3) reading the studies, quality assessment and data evaluation; (4) determining how studies are related; (5) comparing these studies; (6) synthesising studies; and (7) expressing the synthesis.

### 3.2 Getting Started, Rationales for Using Meta‐Ethnography (Phase 1)

We identified no meta‐ethnographic studies in this area, indicating a knowledge gap and a need to synthesise the existing research.

### 3.3 Deciding What Is Relevant, Data Collection—Selection and Search Outcomes (Phase 2)

**FIGURE 1:**
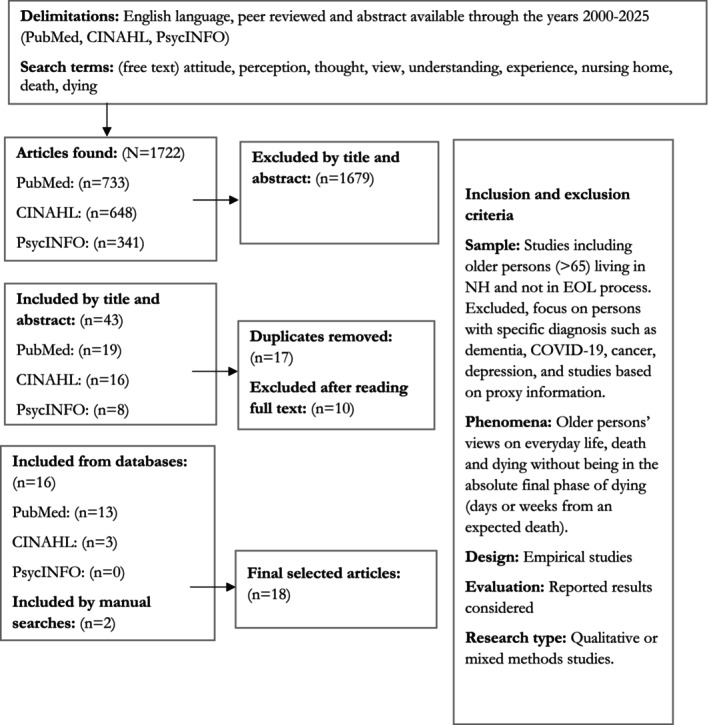
Flowchart of the search strategy.

The selection process followed PRISMA principles for transparency (Figure 1). Only peer‐reviewed studies published in English were included. Duplicate records were identified and removed prior to screening. In collaboration with an experienced librarian, preliminary literature searches were conducted using subject terms in combination with free‐text terms. The preliminary searches indicated that subject terms did not capture the relevant literature, resulting in a decision to proceed with free‐text searches only. The search strategy was restricted to the free‐text term ‘nursing home’. Related terms such as ‘care home’, ‘residential care’ or ‘long‐term care’ were not used as primary search terms due to substantial variation in how these terms are defined across countries and care systems. In particular, the term ‘care home’, commonly used in the United Kingdom, may include settings with or without on‐site nursing care, which was considered to risk reducing specificity in identifying studies relevant to the intended context. To complement the database search, manual searches of relevant references were undertaken, resulting in the identification of additional eligible studies. This reduced the risk of overlooking conceptually relevant studies using alternative terminology. Studies were included based on study‐level eligibility and conceptual relevance to NH life; therefore, studies containing a small number of participants younger than 65 years were retained if the overall sample represented older NH residents.

The systematic research strategy was inspired by the SPIDER tool (Cooke et al. 2012). The final searches and outcomes were conducted in April 2025 and performed in PubMed, CINAHL Complete and PsycINFO using free‐text terms in accordance with Cooke et al. (2012), as this approach can yield the most relevant research results in qualitative research. Studies published between 1 January 2000 and 7 of April 2025 were included, as preliminary searches dating back to 1990 did not identify studies meeting the inclusion criteria. The literature search and study selection were conducted by the first author and discussed with the co‐authors based on the predefined inclusion and exclusion criteria. Disagreements were resolved through consensus. The inclusion criteria comprised qualitative studies involving older persons living in NHs that reported residents' own views on their everyday life, understood as their expressed experiences, attitudes, reflections and meaning‐making related to daily living in the NH context. Everyday life was operationalised as accounts of daily routines, autonomy, social relationships, activities, identity, dignity and the experience of living in NHs.

Studies were included if residents' narratives referred explicitly or implicitly to death and dying, or the proximity of death as part of their everyday life. Explicit discussion of death or dying was not required, provided that such existential dimensions were embedded in residents' accounts of daily living.

While studies including proxy perspectives or mixed‐method designs were eligible for inclusion, the analysis was restricted exclusively to qualitative data derived directly from older persons themselves. In mixed‐method studies, only the qualitative components reporting first‐person narratives of NH residents were included in the synthesis. Case studies and non‐empirical studies were excluded. Studies focusing solely on dying processes, depression, or end‐of‐life care without reference to residents' everyday life were excluded.

### 3.4 Reading the Studies, Quality Assessment and Data Evaluation (Phase 3)

The studies were examined to gain an understanding of their design and structure, evaluated based on the phenomenon of interest and to adopt a critical approach. All included studies had ethical approval. We used the Critical Appraisal Skills Programme (CASP) to assess the methodological quality of the studies. The result of the assessment is presented in Table 1. The CASP checklist was used to highlight the aims in relation to research questions and results, the selection of data collection methods, analysis process and the role of the researchers. The CASP assessments were discussed by the authors. The CASP tool was used to highlight strengths and limitations. No studies were excluded based on methodological quality, in line with Dixon‐Woods et al. (2007), who argues that there is no consensus on exclusion criteria.

**TABLE 1:** Quality appraisal of included studies according to the Critical Appraisal Skills Programme (CASP).

First author, year	1	2	3	4	5	6	7	8	9	10
Bollig et al. (2016)	Yes	Yes	Yes	Yes	Yes	Yes	Yes	Yes	Yes	Yes
Djivre et al. (2012)	Yes	Yes	Yes	Yes	Yes	No	Yes	Yes	Yes	Yes
Dwyer et al. (2008)	Yes	Yes	Yes	?	Yes	No	Yes	Yes	Yes	Yes
Franklin et al. (2006)	Yes	Yes	Yes	Yes	Yes	Yes	Yes	Yes	Yes	Yes
Hall et al. (2009)	Yes	Yes	Yes	No	Yes	No	Yes	Yes	Yes	Yes
Hjaltadóttir and Gústafsdóttir (2007)	Yes	Yes	Yes	Yes	Yes	No	Yes	Yes	Yes	Yes
Hutchinson et al. (2011)	Yes	Yes	Yes	Yes	Yes	No	Yes	Yes	Yes	Yes
Høy et al. (2016)	Yes	Yes	Yes	Yes	Yes	Yes	Yes	Yes	Yes	Yes
Mathie et al. (2012)	Yes	Yes	Yes	Yes	Yes	No	Yes	Yes	Yes	Yes
Oosterveld‐Vlug et al. (2013)	Yes	Yes	Yes	Yes	Yes	No	Yes	Yes	Yes	Yes
Pleschberger (2007)	Yes	Yes	Yes	Yes	Yes	Yes	Yes	Yes	Yes	Yes
Pocock et al. (2021)	Yes	Yes	Yes	Yes	Yes	No	Yes	Yes	Yes	Yes
Scheibl et al. (2019)	Yes	Yes	Yes	Yes	Yes	No	Yes	Yes	Yes	Yes
Shahrki et al. (2018)	Yes	Yes	Yes	Yes	Yes	Yes	Yes	Yes	Yes	Yes
Strohbuecker et al. (2011)	Yes	Yes	Yes	Yes	Yes	No	Yes	Yes	Yes	Yes
Ternestedt and Franklin (2006)	Yes	Yes	Yes	Yes	Yes	?	Yes	Yes	Yes	Yes
Tjernberg and Bökberg (2020)	Yes	Yes	Yes	Yes	Yes	No	Yes	Yes	Yes	Yes
Österlind et al. (2017)	Yes	Yes	Yes	Yes	Yes	No	Yes	Yes	Yes	Yes

*Note:* Yes, No, Can't tell (?) 1 Was there a clear statement of the aims of the research? 2 Is a qualitative methodology appropriate? 3 Was the research design appropriate to address the aims of the research? 4 Was the recruitment strategy appropriate for the aims of the research? 5 Were the data collected in a way that addressed the research issue? 6 Has the relationship between researcher and participants been adequately considered? 7 Have ethical issues been taken into consideration? 8 Was the data analysis sufficiently rigorous? 9 Is there a clear statement of findings? 10 How valuable is the research?

### 3.5 Determining How Studies Are Related, Comparing and Synthesising Studies (Phases 4–6)

The results of the studies were compared and related according to phases four to six as described by France, Uny, et al. (2019). First, the selected studies were read to gain an overview of their content. They were then summarised according to aim, method and results (Table 2).

**TABLE 2:** Characteristics of studies included in the review.

First author, year, country	Objective of the study	Research method	Participants (of relevance for meta‐synthesis)	Results (of relevance for meta‐synthesis)
Bollig et al. 2016, Norway	To study the views of cognitively able residents and relatives on advance care planning, end‐of‐life care and decision‐making in nursing homes.	In‐depth interviews with nursing home residents and focus group interviews with relatives of nursing home residents. Analysis was based on interpretive description.	25 participants living in nine nursing homes. Age: 66–100 years. Mean age 87 years.	Many of the participants had not discussed end‐of‐life and advance care planning with healthcare professionals or relatives, but they expressed views on the future leading up to their inevitable passing. The participants viewed the nursing home as the last stage in life and hoped for a quick and painless death after feeling that they had completed their life journey. The desire to live was connected to health status. When health was good enough to engage in meaningful activities, the desire to live was higher than on days when health was poor, which led to a potential longing for death. Quality of life, for them, involved ending life in a dignified and painless manner, without suffering or unnecessary treatments, surrounded by relatives. They did not wish to become a ‘vegetable’ or be left paralysed.
Djivre et al. 2012, Canada	To investigate older people's (living in a nursing home) experiences of the death of co‐residents and its importance in the development of thoughts about their own death.	Semi‐structured interviews with older adults from a long‐term care home. Hermeneutic‐phenomenological analysis	Five participants living in a nursing home Age: 65–92 years. Mean age 77.6 years.	The demise of fellow residents engendered a sense of emptiness, despite a conscious effort to avoid forming overly close relationships due to the inevitability of impending deaths. Engaging in discussions with other residents about someone's passing served as a means of establishing connections and becoming part of a collective experience larger than individual selves. Additionally, residents evaluated the actions of the staff to gauge whether they, too, would be treated in accordance with their unique personalities and preferences. A prevalent desire among the residents was the need for improved communication with the staff regarding the deaths of fellow residents.
Dwyer et al. 2008, Sweden	To acquire a deeper understanding of how people can create meaning in their everyday life in a nursing home.	Interviews already conducted in the study by Franklin et al. (2006). Analysis with hermeneutic method.	Three women aged 93–95 years living in a nursing home.	Participants expressed a desire for increased social contact, driven by the need to feel valued and a sense of belonging. However, this aspiration was occasionally impeded by the declining functional abilities of co‐residents or their limited family connections. In situations where there were no opportunities for autonomy, the participants' inner resilience became vulnerable to existential threats, leading to a spectrum of responses ranging from acceptance to a desire for death. The inclination towards a death wish seemed to be linked to a perceived lack of belonging.
Franklin et al. 2006, Sweden	To explore the views on dignity expressed by older people living in a nursing home.	Semi‐structured interviews (total 39) over a period of 18 months (Longitudinal study) Analysis was conducted with a hermeneutic approach. These interviews were also used in Ternestedt and Franklin (2006) and Dwyer et al. (2008).	12 people living in one of two nursing homes > 85 years.	The participants had experienced the loss of their own homes. Diminished or lost bodily functions gave rise to feelings of unworthiness, posing a threat to self‐image and identity. Anxiety about the future was prevalent, fuelled by the fear of further loss of functionality. The environment was characterised as passive, and the staff's attitude towards the participants played a crucial role in shaping their self‐image. In response to the threat to their identity, participants endeavoured to compensate by either forging a new sense of dignity or preserving the dignity that endured.
Hall et al. 2009, United Kingdom	To explore the generalisability of Chochinov's dignity model to older people cared for in nursing homes.	Interviews based on ‘Dignity model’. Deductive and inductive content analysis.	18 older people living in one of two care homes with nursing. Age: 78–98 years.	The loss of bodily functions due to old age rendered them dependent on the staff. The nursing home was viewed as the final stage before death, a transition not to be dreaded. As pride diminished, dignity also waned. However, there was a sense of pride associated with maintaining a degree of independence and cognitive abilities. It remained crucial to sustain a semblance of normality, even acknowledging the limitations within a nursing home environment.
Hjaltadóttir and Gústafsdóttir 2007, Iceland	To investigate the characteristics of quality of life of older people living in nursing homes to increase the understanding of the phenomenon of quality of life in this setting.	Interviews over a 3‐month period on two to three occasions. Hermeneutic phenomenology	Eight older people living in one of two nursing homes. Age: 76–93 years.	Participants endeavoured to adapt, aiming to create a sense of home and preserve their individuality within the nursing home setting. While the move to a nursing home was not deemed optimal, the choice often leaned towards security over independence. Life within the nursing home was characterised as monotonous, and any variety of activities was welcomed. Some found a sense of security in the monotony, embracing a philosophy of living day by day. Despite the perceived monotony, there was a shared desire among participants to engage in meaningful activities. Many considered themselves to have completed their life journey and were preparing for the impending reality of death.
Hutchinson et al. 2011, USA	To investigate the personal and environment factors of older people (Caucasians and African Americans) that facilitate adaptation to relocation to long‐term care.	In‐depth interviews. Phase 1 of 3 in a 2‐year study with the aim of developing and testing a cultural heritage intervention to improve adaptation to nursing homes. Longitudinal study Phenomenological analysis.	23 older people from six different nursing homes in the southwestern United States. Age: 60 – > 80 years. Median age: 78.39 years.	Personal factors such as spirituality and belonging to a church were significant influences. The church was also viewed as a form of social belonging. Life experiences and how these were managed also played a role. Participants described feeling a lack of control, even over simple routines such as mealtimes. Life goals diminished, with a simple objective being to move from the bed to the toilet. Participants recognised the need to establish new social connections. They realised that the composition of the staff influenced their sense of control, leading them to strive for compliance so as not to be a burden.
Høy et al. 2016, Denmark, Norway, Sweden	To illuminate the meaning of maintaining dignity in older persons	In‐depth interviews Phenomenological‐hermeneutic method.	28 residents of six different nursing homes in Scandinavia. Age: 62–103 years.	Participants detailed their efforts to preserve dignity despite the loss of bodily functions. These bodily losses had prompted the move to assisted living, heightening vulnerability and a longing for a commonplace existence. Issues that posed challenges at home could be addressed with assistance at the new accommodation, contributing to an enhanced sense of dignity. Simultaneously, a dependency was created since they must rely on assistance. Individuals strive to maintain bodily integrity to the best of their ability, at times refraining from activities they perceive as demeaning and viewing dependency positively (receiving help and being cared for). Social interactions with fellow residents, especially during mealtimes, held significant importance, contingent upon their physical capabilities. Assistance was perceived as both a source of strengthened self‐esteem and a potential threat, with losses not impeding this duality. They expressed a desire to be acknowledged as individuals and to actively participate in decision‐making processes.
Mathie et al. 2012, United Kingdom	To explore the views, experiences and expectations of end‐of‐life care among care homes residents to understand if key events or living in a residential environment influenced their views.	The design used a mixed‐methods approach, with interviews conducted longitudinally over 1 year. Thematic analysis was used.	121 people from six different care homes (90 completed). Age: 61–102 years. Average age: 87.5 years.	A limited number of participants altered their perspectives on end‐of‐life matters over the course of the study. Instances occurred where participants shifted between different frameworks within a single interview. Satisfaction with residing in residential care was not universal; some expressed a desire to return home, while others lamented their dependency on care, realising that returning home was not feasible. Contemplating the future proved challenging for participants due to its inherent uncertainty. While they acknowledged the necessity of living in a care home, it engendered a sense of limited control over their lives and, consequently, their futures. This perceived lack of purpose led to a yearning for death. Participants expressed a preference for a natural death over resuscitation and indicated a preference to stay in the care facility rather than be transferred to a hospital.
Oosterveld‐Vlug et al. 2013, Netherlands	To investigate if and how the dignity of nursing home residents´ changes over the course of time, and what contributes to this.	Multiple in‐depth interviews with an interval of 6 months. Longitudinal study Thematic analysis.	22 people from four different nursing homes. Age: 49–97 years. Mean age: 77 years. Three people ≤ 60 years.	Participants conveyed a sense of longing for their homes and familiar routines, yet they acknowledged and accepted their current situation. Clear communication with healthcare staff regarding preferences, such as shower routines and dining locations, facilitated a more comfortable environment. The participants grappled with feelings of worthlessness stemming from the loss of function and dependence on others. Even minor health improvements or the preservation of existing functions held considerable significance. The participants articulated the ongoing struggle to uphold their social status in the face of these challenges.
Pleschberger 2007, Germany	To explore the meaning of dignity regarding end‐of‐life issues from the perspective of older nursing home residents in Western Germany.	The study was part of a larger study. Narrative interviews were conducted, and analysis was based on Grounded Theory.	20 people living in six different nursing homes. Age: 63–93 years. Mean age: 82 years.	The term ‘dignity’ did not emerge directly from the participants' expressions. However, family relationships were emphasised as being crucial for well‐being. Ageing alone does not inherently confer high dignity, but the essence of life is perceived as an ongoing quest to attain dignity despite advancing age. One strategy involves not being a financial or decision‐making burden on the next generation. The heightened need for assistance poses a threat to dignity, as it directly impacts one's body and, consequently, one's sense of identity. The challenge intensifies with each functional loss, with dementia looming as a substantial threat. Participants mourned the loss of social connections and harboured fears of burdening their relatives, experiencing dementia, losing functions and becoming dependent on others. A dignified death, in their view, is one that occurs at the right time—before reliance on others becomes too pronounced. The prospect of no longer being able to perform tasks independently evoked a sense of horror.
Pocock et al. 2021, United Kingdom	To give voice to those in the fourth age, learning about their perspectives on transitions to, and life and death within, care homes.	Interviews based on subject guides, a total of 19 interviews over a 10‐month period. Longitudinal study Structured narrative analysis.	Five people living in two different nursing homes. Age: 86–96 years.	The participants did not perceive the place they lived in as their own home, and they experienced a tension between autonomy and security, with an inclination towards accepting safety and security. There was a poignant expression of sadness associated with dependency, and a realisation of being a burden that needed to be alleviated. The desire not to be resuscitated and the notion of death during sleep as a preferable outcome were articulated as elements of a ‘good death’. They felt a lack of involvement in the decision to move from their home to the new accommodation. Participants described efforts to present themselves as compliant and positive residents. While death was viewed as inevitable, it was not openly discussed, revealing a certain ambivalence towards the topic.
Scheibl et al. 2019, United Kingdom	To provide a typology of the experiences of people in very old age of moving to a nursing home that is clinically useful for practitioners navigating very old people's relocation	Qualitative analysis of interview data from a mixed‐methods UK population‐based longitudinal study to identify the experiences of moving to a nursing home. Thematic analysis.	26 older people. Age 95–100 years. Mean age: 97.3 years.	Relatives played a significant role in the well‐being of the participants. Those with limited contact with family expressed a sense of having nothing to live for. The act of moving was viewed as altruistic, a way to alleviate the perceived burden on loved ones. Despite accepting a higher level of dependence, participants acknowledged that it brought a sense of security. A common complaint was the lack of social interaction with other residents. There was a notable absence of communication and meaningful conversations. The participants lamented the loss of freedom and privacy that they would have had in their own homes. Nevertheless, they accepted these limitations in exchange for the priority of security. Seeking the company of others remained a desire if possible.
Shahrki et al. 2018, Iran	To explore the experiences of older residents who are dying in nursing homes and their caregivers as well as attitudes towards death in an Iranian context.	Approach used was focused ethnography. Observations, semi‐structured interviews, field notes. Thematic analysis.	Ten older people from two semi‐private mixed‐sex nursing homes. Age: 65–85 years.	The participants anticipated a peaceful death devoid of pain. With ageing seen as a diminishing of value, death was perceived as a liberator from the challenges of life. There was a reluctance to being a continuing burden on family and relatives, with a perception that loved ones might wish for their passing. Participants did not express mourning for those who had died, since they believed that the happiness derived from those deaths contributed to an enhancement in the overall quality of life.
Strohbuecker et al. 2011, Germany	To explore the palliative care needs of nursing home residents in Germany who had not yet entered the dying phase.	Semi‐structured interviews. Grounded theory approach in analysis.	Nine residents suffering from chronic disease or frailty living in a nursing home. Age: 70–100 years. Mean age: 86.5 years.	The participants expressed a strong desire to be recognised as individuals with unique histories, habits and preferences. Physical ailments had diminished their control over daily activities, and they yearned for more autonomy, particularly in decisions related to food, hygiene and the ability to go out. However, they sometimes refrained from sharing their wishes, recognising that they might not be fulfilled. The greatest fear among participants was the prospect of becoming bedridden and increasingly dependent. Family held significant importance, and they remained curious about events in the immediate outside world. A priority for them was to be pain‐free, and they approached death without excessive worry. Some participants advocated for more openness about death and dying. They expressed a preference for minimal treatment and a reluctance to be kept alive unnecessarily.
Ternestedt and Franklin 2006, Sweden	To reach a deeper understanding of the thoughts and feelings of some older people living in a nursing home and how they relate to life close to death.	The study was part of a larger project reported in Franklin et al. (2006). Interviews were conducted on four occasions over a period of 18–24 months. Seven out of the first sample of 12 nursing home residents were included. A hermeneutic design was used for analysis.	Seven older persons living in a nursing home aged > 86 years.	The participants acknowledged the inevitability of death but remained committed to staying active. While prepared for death, they still contemplated experiencing certain things before that time. As dependence increased, life became more restricted, yet they derived positivity from the experiences gained over their lifetimes. Security took precedence over independence, although it did not fully replicate the feeling of living at home. Frustration emerged from an inability to participate in the planning of their care and daily activities. The perception of diminishing value with ageing led to a yearning for death as a liberator. It was essential to maintain an image of a past life as healthy and, if feasible, to continue with activities from that period. Loss of function and a lack of social relationships could render life seemingly meaningless, potentially leading to a desire for death. On the other hand, some participants found satisfaction in their lives, considering themselves to have fulfilled their life's journey.
Tjernberg and Bökberg 2020, Sweden	To explore thoughts about death and dying and experiences of care at the end‐of‐life among older persons living in nursing homes.	Individual interviews conducted by RN's following an interview guide allowing spontaneous answers. Content analysis according to Graneheim and Lundman.	36 persons living in different nursing homes. Age: 67–102 years. Mean age: 88 years.	The participants struggled to position themselves in the final phase of life. Although the future was perceived as uncontrollable, it did not evoke fear, since they felt they had lived their lives. Meaning was derived from relationships with children and grandchildren, being of significance to someone else brought a sense of purpose. There was, however, a fear of a slow and painful death, and a desire to remain healthy until the end. The nursing home was viewed as a guarantee of security. Death was seen as a release from a deteriorating body, with lost bodily functions instigating fears of becoming a burden. Increased dependence resulted in fewer activities and heightened boredom. The loss of function produced a perceived loss of independence and control, prompting comparisons with a past life and a recognition of transformation. Participants expressed a desire to exert some control over death, to be prepared and to bid farewell to their families. Living with others was stressful since they perceived fellow residents as being more sick and having greater care needs
Österlind et al. 2017, Sweden	To deepen the understanding of how older persons living in a nursing home experience life close to death	Interviews were conducted between one and four times (total number = 16). Interpretation process inspired by Gadamer.	Six older people in four nursing homes. Age: 77–97 years.	The participants expressed trying to cope with their lives, finding reassurance in the proximity of staff for assistance. Simultaneously, they mourned the loss of independence and the inability to reside in their own homes. They were acutely aware of the brevity of their future, considering it a natural part of a long life. Despite this, conversations about death with staff or relatives were infrequent. Participants perceived time as a waiting period for death, seen as a relief from pain, difficulties and negative emotions. Adjusting to the routines of the facility was seen as an intrusion on their identity. Some participants desired more influence and criticised the fact that their physical limitations placed them in the hands of staff. The lack of activity led to a sense of meaninglessness. Coming to terms with the new situation proved challenging, and participants desired to be treated in a way that was based on their past selves. The lack of consideration for their perspectives left participants feeling diminished, leading them to employ various strategies to maintain their identity.

In Phase 4, the studies were examined in relation to each other in terms of purpose and context, determining how they were connected and which aspects were compared. The outcome was presented as descriptive themes, closely related to the text, such as losses of different kinds, views and experiences of death and dying, and everyday life (Table 3). First‐order constructs (participants' accounts) and second‐order constructs (authors' interpretations) were compared across studies to identify recurring meanings and concepts. In Phase 5, the studies were compared and interpreted with a focus on reciprocal aspects, leading to the organisation of conceptual categories and interpreting them thematically. The comparisons were formulated as analytical themes, such as *Loss of the familiar world* (Table 3). Finally, in Phase 6, the themes were synthesised and presented as an overarching metaphor: *Maintaining and shaping a living space for a visible future*. The overarching metaphor emerged from the analytical themes developed through translation and comparison across studies. Table 3 provides a visual overview of the meta‐ethnographic analytic progression, illustrating how descriptive themes (Phase 4) were translated into analytical themes (Phase 5) and synthesised into an overarching metaphor (Phase 6). The final phase (Phase 7), expressing the synthesis, is presented in the Discussion.

**TABLE 3:** Meta‐ethnographic analytic progression from descriptive themes to analytical themes and overarching metaphor.

Phase 6 ‐ Overarching metaphor: Maintaining and shaping a living space for a visible future		
Phase 5 – Analytical themes	Phase 4 – Descriptive themes	Illustrative quotes (examples)
Loss of the familiar world	Losses of home	Of course you miss your home and all the books and the things you have collected all your life…this [moving to the nursing home] was the next best thing. (Hjaltadóttir and Gústafsdóttir 2007, 51)
	Losses of meaning, value, belonging	As soon as we get old and cannot manage our personal affairs, we lose our worth. I swear to God that death now is a divine blessing… (Shahrki et al. 2018, 507)
	Fear of future losses	And that is a horror for me, that I might possibly have to lie in bed from morning to evening…that I am dependent on another person to make every handhold that is important for my care and support. (Pleschberger 2007, 200)
	Functional losses. Decreased abilities	I can't get in the bath, so they wash me down every morning in here…That to me is embarrassing but you got to accept it…I can't do anything for myself, or very little… Depending on people and I don't want that’. (Hall et al. 2009, 413)
	Dependence of health care staff and nursing home routines	…it feels as if they treat us as if we don't understand anything even though we have lived a whole life…everything is supposed to be done quickly and fit into the right box. We are told when to eat, when to shower… (Franklin et al. 2006, 139)
Trying to maintain personal identity	Striving for independence in activities	Do you know what makes me happy? To be able to go to the toilet on my own. It gives me satisfaction not having to ask for help with everything. I'm so grateful that I can take care of myself as much as I can. (Dwyer et al. 2008, 101)
	Acceptance/dignity	Then [when I had just arrived in the nursing home] I felt I had lost some of my dignity…I didn't know how things worked, so I felt inferior…Then you realize you aren't inferior to the rest and then it comes back. (Oosterveld‐Vlug et al. 2013, 5)
Death as close and tangible	Death as natural, acceptance, respect	When you are younger, you want to live a bit longer. You're old – well then your time has passed. Like me now, I'm okay, I can go at any time I want… (Djivre et al. 2012, 503)
	Longing or waiting for death	I don't want to get up. I want to die now. I don't have a lot of strength left. It would be nice to leave this earth, that'show it feels…it feels nice; it has for a long time. (Ternestedt and Franklin 2006, 338)
	Wishes of a painless death	…Either a stroke or a heart attack…unless I'm one of these – when you hear of people dying in their sleep or anything. But that doesn't happen – I wouldn´t think that happens as often as a stroke (Pocock et al. 2021, 1646)
	Concluding life, both bad and good. Striving for satisfaction	I'm not keen on this long life that everyone is striving for…I admire life, it has given me many things, both hard work and also happiness and I can only say that I try as hard as I can to live because life has been given to me. (Hjaltadóttir and Gústafsdóttir 2007, 52)

## 4 Results

### 4.1 Contextual Information of Included Studies

The study selection process is illustrated in the PRISMA flow diagram (Figure 1). Following full‐text screening, 18 studies were included in the synthesis and the characteristics of the studies are presented in Table 2. In addition, 10 full‐text articles were reviewed but excluded based on the predefined exclusion criteria. Hall et al. (2009) and Scheibl et al. (2019) were identified through manual searches. Dwyer et al. (2008), Franklin et al. (2006) and Ternestedt & Franklin et al. (2006) were based on the same data collection. The studies had been conducted in Europe (*n* = 15), Canada, Iran and the United States.

All studies explored the experiences and views of older persons residing in NHs on everyday life and their reflections on their approaching death and dying. Participants described adapting to NH life, comparing past and present and considering their future. Some studies addressed dignity and meaning, while others focused on transitions and cultural or personal influences. The age of the participants in these studies varied widely, ranging from 49 to 103 years, highlighting the diverse age spectrum of individuals living in NHs. Details for all studies are presented in Table 2 and, for studies including informants younger than 65 years, in Table 4. Overall, the studies were of high quality, with the most common shortcoming being insufficient consideration of the relationship between the researcher and the participants, rated as ‘no’ or ‘unclear’ in 13 of the 18 studies. This limitation may have influenced how participants' experiences were expressed and interpreted, particularly regarding sensitive topics. This potential limitation was addressed in the synthesis by focusing on first‐person accounts and identifying recurring patterns across studies from different contexts.

**TABLE 4:** Characteristics of studies with participants < 65 years.

First author, year	Total participants, age range	Mean age
Hutchinson et al. (2011)	*n* = 23 60–70 years: *n* = 7 71–80 years *n* = 6 > 80 years *n* = 10	Unreported
Høy et al. (2016)	*n* = 23 62–103 years	Unreported
Mathie et al. (2012)	*n* = 121 61–102 years	87.5 years
Oosterveld‐Vlug et al. (2013)	*n* = 22 49–97 years ≤ 60 *n* = 3 61–70 *n* = 3	77 years
Pleschberger (2007)	*n* = 20 63–93 years	82 years

### 4.2 Findings

We have chosen to present the findings in three themes: *Loss of the familiar world, Trying to maintain personal identity, Death as close and tangible*. The themes presented below were developed through iterative comparison and synthesis across the included studies (see Methods and Table 3).

#### 4.2.1 Loss of the Familiar World

Across the included studies there were recurrent expressions of a profound sense of loss regarding the familiar world. Participants described losses encompassing their physical bodies, homes, friendships, family connections and views about the future. In some studies participants expressed fears of additional losses, such as cognitive impairment, becoming bedridden and an increased dependence on others (Bollig et al. 2016; Franklin et al. 2006; Hall et al. 2009; Pleschberger 2007; Strohbuecker et al. 2011; Tjernberg and Bökberg 2020).

One person conveyed how the loss of hearing and vision had altered the way he was treated, making him feel dehumanised and reliant on the staff to maintain social contacts (Franklin et al. 2006). Social relationships with other co‐residents could be perceived as forced and devoid of meaning, as communication was hindered by co‐residents' disabilities (Dwyer et al. 2008; Hjaltadóttir and Gústafsdóttir 2007; Høy et al. 2016; Tjernberg and Bökberg 2020). Finding social affiliation with others was something that could enhance well‐being, but also risked evoking thoughts of mortality, since co‐residents were described as frail and at risk of passing away (Djivre et al. 2012; Høy et al. 2016).

Across the studies, residents' losses of bodily functions combined with negative care experiences were associated with expressions of alienation. The disconnect between the care they needed and the care they received deepened their sense of isolation from their environment and those around them (Djivre et al. 2012; Dwyer et al. 2008; Franklin et al. 2006; Hjaltadóttir and Gústafsdóttir 2007; Hutchinson et al. 2011; Høy et al. 2016; Mathie et al. 2012; Pleschberger 2007; Oosterveld‐Vlug et al. 2013; Pocock et al. 2021; Strohbuecker et al. 2011; Tjernberg and Bökberg 2020; Ternestedt and Franklin 2006; Österlind et al. 2017). As a result, in some studies, participants experienced a profound sense of diminished value and meaning in their lives (Franklin et al. 2006; Hall et al. 2009; Høy et al. 2016; Mathie et al. 2012; Oosterveld‐Vlug et al. 2013; Pleschberger 2007; Ternestedt and Franklin 2006).

In some studies, participants described how they felt forced to move from their previous home to a NH and were not involved in the decision‐making process (Pocock et al. 2021; Strohbuecker et al. 2011). The presence of staff made it difficult to maintain a sense of integrity, while a shortage of staff made it difficult for them to get help when they needed and wanted it, or go outside (Djivre et al. 2012; Franklin et al. 2006; Hjaltadóttir and Gústafsdóttir 2007; Hutchinson et al. 2011; Høy et al. 2016; Mathie et al. 2012; Oosterveld‐Vlug et al. 2013; Pocock et al. 2021; Scheibl et al. 2019; Strohbuecker et al. 2011; Ternestedt and Franklin 2006; Tjernberg and Bökberg 2020; Österlind et al. 2017).

#### 4.2.2 Trying to Maintain Personal Identity

Across the studies participants described how they made extensive efforts to maintain their personal identity through various means. Participants in several studies described striving to retain their independence as much as possible by drawing on internal resources, staying connected with their families, engaging in social interactions and utilising support from the staff (Djivre et al. 2012; Dwyer et al. 2008; Franklin et al. 2006; Hall et al. 2009; Hjaltadóttir and Gústafsdóttir 2007; Hutchinson et al. 2011; Høy et al. 2016; Mathie et al. 2012; Oosterveld‐Vlug et al. 2013; Pocock et al. 2021; Scheibl et al. 2019; Ternestedt and Franklin 2006; Tjernberg and Bökberg 2020; Österlind et al. 2017). One woman stressed the importance of being able to go to the bathroom on her own (Dwyer et al. 2008).

In one study, a participant described how being able to manage their wheelchair independently was sufficient to preserve personal identity (Oosterveld‐Vlug et al. 2013). One woman expressed gratitude towards her daughter, who regularly took her out for walks. This assistance enriched her well‐being, helping her to stay connected to the outside world and maintain a sense of normalcy in her life (Tjernberg and Bökberg 2020).

Striving for meaningful relationships with family, friends, staff and other residents emerged as a significant component in maintaining identity and self‐worth (Djivre et al. 2012; Dwyer et al. 2008; Franklin et al. 2006; Hall et al. 2009; Hjaltadóttir and Gústafsdóttir 2007; Hutchinson et al. 2011; Høy et al. 2016; Mathie et al. 2012; Oosterveld‐Vlug et al. 2013; Pocock et al. 2021; Scheibl et al. 2019; Strohbuecker et al. 2011; Tjernberg and Bökberg 2020; Österlind et al. 2017). In some studies, participants described how they appreciated the move to a NH, since it offered opportunities for social activities and involvement in various events. Helping other residents, especially during mealtimes, gave them a sense of purpose and contribution (Franklin et al. 2006; Høy et al. 2016; Oosterveld‐Vlug et al. 2013; Ternestedt and Franklin 2006; Österlind et al. 2017).

Participants in several studies highlighted active engagement in activities and nurturing personal interests as crucial for maintaining their sense of identity (Dwyer et al. 2008; Hjaltadóttir and Gústafsdóttir 2007; Hutchinson et al. 2011; Høy et al. 2016; Oosterveld‐Vlug et al. 2013; Strohbuecker et al. 2011; Ternestedt and Franklin 2006; Tjernberg and Bökberg 2020; Österlind et al. 2017). A woman expressed her desire to continue her interest and stated that she could not just lie there and think that she could not do anything (Österlind et al. 2017). Another woman expressed her desire to show others how to embroider (Ternestedt and Franklin 2006). However, Høy et al. (2016) noted that some older persons preferred to avoid activities that reinforced their retired status, despite staff attempts to involve them in past interests, such as baking. Standing up for themselves and communicating their preferences to the staff reinforced their sense of value and dignity.

#### 4.2.3 Death as Close and Tangible

Across the included studies, death emerged as a close and tangible presence in the participants' accounts. Participants expressed reflections on their future death and viewed it as something natural and expected, sometimes even wishing for it (Bollig et al. 2016; Djivre et al. 2012; Hall et al. 2009; Mathie et al. 2012; Shahrki et al. 2018; Strohbuecker et al. 2011; Ternestedt and Franklin 2006; Tjernberg and Bökberg 2020; Österlind et al. 2017). Death itself was often not perceived as frightening; it was the process of dying or becoming bedridden and dependent on others that caused concern, leading to a desire for a death free from suffering (Bollig et al. 2016; Hall et al. 2009; Mathie et al. 2012; Pleschberger 2007; Pocock et al. 2021; Shahrki et al. 2018; Ternestedt and Franklin 2006; Tjernberg and Bökberg 2020).

In some studies, older persons expressed a desire to die, however, this varied depending on their physical and mental health on the day (Bollig et al. 2016). For others, this reflected a more deep‐rooted wish to die, expressed as ‘It seems to me quite non‐sensible to go on existing as I am’ (Mathie et al. 2012). Death was often perceived as something close and tangible, partly because friends of the same age began to disappear (Djivre et al. 2012; Dwyer et al. 2008). It was difficult to plan life, so they lived in the present (Bollig et al. 2016; Mathie et al. 2012).

In some studies, participants reported seeing no point in continuing to live. Participants in several studies described having no contact with relatives or no one to talk to and perceived their lives as essentially complete. What remained was remembering the past (Bollig et al. 2016; Djivre et al. 2012; Dwyer et al. 2008; Hjaltadóttir and Gústafsdóttir 2007; Shahrki et al. 2018; Ternestedt and Franklin 2006; Tjernberg and Bökberg 2020; Österlind et al. 2017).

A calm and peaceful death, surrounded by relatives or trusted individuals, was described as desirable by some participants. Dying in the presence of strangers was not perceived as positive (Ternestedt and Franklin 2006). Others had considered how they should prepare for their death and funeral to make it easier for their relatives (Pleschberger 2007; Strohbuecker et al. 2011; Ternestedt and Franklin 2006; Tjernberg and Bökberg 2020; Österlind et al. 2017).

A recurring pattern across studies was that participants in several studies expressed feeling content with the way they had lived their lives despite life's ups and downs. When reminiscing about past life events, participants in some studies acknowledged and embraced both the enjoyable and less enjoyable moments (Bollig et al. 2016; Dwyer et al. 2008; Hjaltadóttir and Gústafsdóttir 2007; Hutchinson et al. 2011; Scheibl et al. 2019; Ternestedt and Franklin 2006; Tjernberg and Bökberg 2020). Age brought a sense of stability, leading to a desire to reflect on life, including both the mistakes made and moments cherished, fully aware that nothing about the past could be altered (Dwyer et al. 2008; Hjaltadóttir and Gústafsdóttir 2007).

Remembering their younger years could be a component in summarising and being content with life as one person expressed: ‘I have lived my life my own way, with friends and lots of travelling.’ (Dwyer et al. 2008). They viewed life through a retrospective lens, connecting it to the future through future generations. One woman conveyed this sentiment with the words, ‘Look at these pictures. It is my child, grandchild and her newborn. Can you believe, we are four generations.’ (Franklin et al. 2006). In the studies, expressions of preparedness for death coexisted with continuing desires to live.

Across the findings, loss of the familiar world emerged as a catalyst for participants' efforts to maintain personal identity. The proximity of death shaped these strategies by narrowing—but not eliminating—a sense of future, influencing how meaning and dignity were experienced in everyday life.

## 5 Discussion

Based on the findings of this meta‐ethnographic study, we propose that older persons' views on everyday life, and reflections on future death and dying in NH can be understood through the overarching metaphor of *maintaining and shaping a living space for a visible future*. This overarching metaphor links the three themes and explains how everyday life in NHs unfolds over time and through relationships.

Loss, identity formation and the proximity of death are not separate experiences. Instead, they are closely interconnected processes through which older persons strive to maintain dignity, meaning and a sense of the future, despite limitations in their situation. At the level of synthesis, death and dying are understood not simply as signs of approaching death, but as reflections that are integrated into everyday meaning‐making and the ageing process. These findings have clear implications for older people nursing, highlighting the importance of addressing identity, dignity and existential concerns as part of everyday nursing care in NHs.

### 5.1 Overarching Metaphor—Maintaining and Shaping a Living Space for a Visible Future

Everyday life in a NH is shaped by reflections on the past, experiences of loss in the present, and expectations of a limited future. In the context of NHs, older persons strive for independence and draw on personal resources. They also engage in relationships to express and preserve their identity in everyday life and in the proximity of death and dying.

In line with Nordenfelt's concept of dignity as identity (Nordenfelt 2021), the findings show that dignity is shaped in relationships and can be maintained when individuals are recognised as autonomous persons with a past and a future. In this study, we suggest that *loss of the familiar world* comprises the loss of personal routines and self‐worth. This loss poses a threat to older persons' striving to maintain and shape a living space for a visible future. Öhlén et al. (2014) described how losses can affect older persons' health and well‐being. They use the metaphor of the polar extremes of ‘at‐homeness’ and ‘homelessness’. In this view, at‐homeness represents a state of well‐being despite illness, and homelessness signifies an inability to attain this state. For older persons, the struggle for at‐homeness can influence their outlook on the future, often forcing them to accept their circumstances rather than advocate for themselves or initiate change where possible. This struggle frequently involves drawing on their functional abilities, expressing preferences in daily activities and preserving their autonomy.

The findings of this study show how the loss of a familiar world impacts personal dignity and significant relationships with others. These findings can be related to previous research, such as that of Søvde et al. (2022) who highlighted the risk of losing a sense of ‘at‐homeness’ in institutional care. The authors reported that the older persons described home as a shelter where they could feel safe. At the same time, the loss of bodily functions made their home feel unsafe, which led the older persons to seek to regain a sense of at‐homeness within the new experience of living in an institution.

From this perspective, the loss of the familiar world can be seen as a shrinking of the living space. This threatens both everyday life and the sense of a future, making efforts to maintain a meaningful life more fragile and urgent.

As demonstrated in this study, the loss of bodily functions may impair social interactions with co‐residents or staff, but can also motivate older persons to maintain their identity by regaining a sense of at‐homeness within institutional boundaries. Based on our findings, we suggest that *trying to maintain personal identity* may involve adopting a ‘one day at a time’ attitude, viewing death as an inevitable, yet acceptable, part of life. Alternatively, older persons may take advantage of every opportunity to influence their situation. Paddock et al. (2019) highlighted that older persons in NHs employ adaptive strategies, such as social comparison with residents with dementia, to maintain a sense of being a person with superior cognitive ability. These strategies can be regarded as expressions of their identity. Acceptance of the situation aligns with maintaining their original identity or forging a new one. In both cases, a sense of the future remains, either as a longing for death or as a desire to continue shaping everyday life. According to Öhlén et al. (2014), at‐homeness can, despite illness, encompass being acknowledged as a person and feeling connected to others, finding safety through the attainment of meaning and experiencing comfort and harmony by recognising one's inner self. It is a process of moving from a sense of homelessness towards achieving at‐homeness, which can be understood as part of the process of shaping personal identity in line with the realm of *dignity of identity* as described by Nordenfelt (2021). In this study, this approach was evident in older persons' attempts to engage in activities they used to enjoy, or in interest in teaching others needlework. Supporting identity therefore requires allowing room for everyday agency, recognising personal preferences and valuing older persons' contributions—even in small, ordinary situations. This may involve offering opportunities for everyday choices, facilitating conversations about life experiences, existential issues and future hopes and concerns and supporting participation in meaningful activities. For nursing staff, this requires flexibility in everyday routines and responsiveness to NH residents' individual preferences.

These efforts to maintain personal identity can be seen as active attempts to expand and inhabit a meaningful living space. In this way, a future—although limited—remains visible in everyday life. In the context of this synthesis, *death as close and tangible* forms the existential horizon within which everyday life is lived, and the living space is continually shaped. Awareness of approaching death limits the future but does not remove it. Rather, the visible future becomes shorter and more relational, shaping how older persons invest meaning in everyday interactions, memories and relationships. Identity formation thus involves either projecting oneself into future generations or reconciling with a finite future through reflection on a lived life. For care practice, this underscores the importance of legitimising conversations about death and dying as part of everyday life in NHs rather than treating such expressions as solely pathological or problematic.

Contemplating death is therefore understood not as withdrawal, but as an identity‐shaping process closely tied to relational engagement, as supported by Shin (2015), who underscores the significance of relational connections in sustaining identity in the face of mortality. From an existential point of view, death is deeply intertwined with identity formation through the capacity to foster and maintain meaningful relationships. In this way, the proximity of death intensifies rather than diminishes the ongoing work of maintaining and shaping a living space for a visible future. The capacity to foster meaningful relationships is emphasised in experimental research conducted by Blackie et al. (2016) and described by Coleman (1989) as a crucial component in fostering identity in old age. Within relational contexts, older persons can find continuity and meaning as life approaches its end. An active social life and meaningful relationships are crucial for sustaining dignity, identity and a sense of continuity when death is close (Bruggencate et al. 2018).

As individuals age, the fear of losing friends becomes more palpable, particularly as they confront the possibility of their own serious illness or mortality. Moreover, the loss of physical function may limit their capacity to maintain or cultivate new social relationships (Hilari and Northcott 2017). The findings in this study corroborate the idea that functional losses and the possible absence of family relationships ultimately increase their reliance on NH staff. Functional losses and institutional routines may constrain everyday life and increase reliance on NH staff. However, reciprocal relationships and opportunities for agency enable older persons to sustain belonging and autonomy.

This synthesis shows that care relationships play a key role in whether everyday life becomes more limited or more meaningful when death is close. As losses of the familiar world increase vulnerability, healthcare professionals play a pivotal role in supporting the dignity of identity through recognition, autonomy and reciprocal relationships (Moilanen et al. 2022; Eldh et al. 2016). However, care practices shaped by compassionate ageism risk narrowing this living space by positioning older persons as dependent and passive, thereby undermining identity and reinforcing alienation (Levy 2009). In contrast, person‐centred and relational care can sustain belonging, agency and continuity by supporting dignity of identity, thereby enabling older persons to maintain and shape a meaningful living space for a visible future (Rejnö et al. 2020).

Taken together, this synthesis indicates that NH care should be understood as an ongoing relational practice shaping older persons' opportunities to maintain dignity, identity and a visible future, even when death is close. The themes reflect shared interpretive patterns across diverse experiences rather than uniform perspectives.

### 5.2 Methodological Reflections

In line with recommendations for meta‐ethnographic synthesis, the number of included studies was limited to allow interpretive depth. Eighteen studies were considered sufficient for this purpose.

The database searches were based solely on the term ‘nursing home’. While this may be considered a limitation in an integrative review, it was judged most appropriate as it reflects settings providing both personal care and nursing by registered nurses. Broader terms such as ‘care home’, ‘residential care’ or ‘long‐term care’ were excluded due to inconsistent definitions or the retrieval of large numbers of irrelevant studies. While ‘care home’ is commonly used in the United Kingdom, its variable application across contexts limited its usefulness as a primary search term. Three included studies did use the term ‘care home’; however, these settings provided comprehensive personal care for older persons with complex needs, and the findings were conceptually transferable to NH contexts. Excluding these studies would have risked omitting relevant evidence on key phenomena of interest. Their inclusion was therefore justified based on conceptual rather than terminological alignment.

Our intention was to include studies involving persons aged over 65 years; some variation occurred. In five studies, a small number of participants were younger than 65 years; however, the overall sample (381 participants) predominantly comprised older persons, with a mean age above 75 years (Table 4). This variation was not considered to substantially influence the findings, but it is acknowledged as a limitation, as younger participants may have different illness trajectories and life situations. More broadly, residents in NHs constitute a highly heterogeneous group, and the themes should be understood as interpretive patterns across diverse experiences rather than a uniform perspective.

The included studies mainly represent older persons who were cognitively able to participate in interviews. Therefore, the perspectives of residents with more advanced cognitive impairment or dementia are less visible in this synthesis, highlighting an important gap in the literature. As a result, the synthesis primarily reflects verbally articulated experiences and may not capture how these existential dimensions are expressed among NH residents with limited verbal communication.

A common methodological limitation across the included studies was the limited consideration of the researcher–participant relationship, which may have influenced how residents' views were expressed and interpreted. This was addressed in the synthesis by focusing on first‐person accounts and by comparing recurring patterns across studies from different contexts.

## 6 Conclusion

This meta‐ethnographic synthesis demonstrates that everyday life in NHs is shaped by intertwined experiences of loss, identity work and an ongoing awareness of death and dying. Rather than signalling disengagement, the proximity of death forms an existential horizon within which older persons actively strive to uphold dignity, meaning and a sense of self. Through the overarching metaphor of maintaining and shaping a living space for a visible future, the synthesis highlights how everyday interactions, autonomy and relationships remain central to identity formation even in the final stages of life. However, it should be noted that the synthesis primarily reflects the perspectives of residents who were able to participate in interviews and may therefore not fully represent residents with more advanced cognitive impairment. Supporting older persons' identity should be understood as an essential relational dimension of everyday nursing care.

## Author Contributions

**Peter Lundberg:** methodology, data collection, synthesis, writing (original draft, review). **Jane Österlind:** supervision, synthesis, writing (review, editing, validation). **Malin Olsson:** supervision, synthesis, writing (review, editing, validation).

## Ethics Statement

The authors have nothing to report.

## Conflicts of Interest

The authors declare no conflicts of interest.

## Supporting information


**Data S1:** opn70097‐sup‐0001‐Supinfo1.docx.

**Data S2:** opn70097‐sup‐0002‐Supinfo2.docx.

## Data Availability

All data analysed during this study are available in the published articles cited in this manuscript. No new data were generated.
